# Mediating effects of physical activities and cognitive function on the relationship between dietary diversity and all-cause mortality in community-dwelling older adults

**DOI:** 10.7189/jogh.14.04169

**Published:** 2024-10-25

**Authors:** Chi Zhang, Anying Bai, Guoqing Fan, Ji Shen, Yuting Kang, Pengjun Zhang

**Affiliations:** 1 The Key Laboratory of Geriatrics, Beijing Institute of Geriatrics, Institute of Geriatric Medicine, Chinese Academy of Medical Sciences, Beijing Hospital, National Centre of Gerontology of National Health Commission, Beijing, China; 2 School of Population Medicine and Public Health, Chinese Academy of Medical Sciences and Peking Union Medical College, Beijing, China; 3 Department of Geriatrics, Beijing Hospital, National Centre of Gerontology, Institute of Geriatric Medicine, Chinese Academy of Medical Sciences, Beijing, China; 4 Department of Science Research, Beijing Hospital, National Centre of Gerontology, Institute of Geriatric Medicine, Chinese Academy of Medical Sciences, Beijing, China

## Abstract

**Background:**

Although dietary diversity (DD) has been confirmed to be associated with multiple health outcomes and longevity in older people, the related mechanisms have not been elucidated. In this study, we explored the mediating roles of physical activities and cognitive function in the relationship between DD and all-cause mortality.

**Methods:**

We recruited 34 068 community-dwelling older adults aged ≥60 years from the Chinese Longitudinal Healthy Longevity Study and followed them up until 2018. Dietary diversity score (DDS) was assessed by the intake frequency of nine food sources. We evaluated physical activities and cognitive function using the Katz index and Mini-Mental State Examination. We explored the mediating roles of physical activities and cognitive function between DDS and all-cause mortality using mediated analyses in Cox proportional risk regression models.

**Results:**

A total of 25 362 deaths were recorded during 148 188.03 person-years of follow-up. Participants with physical disability and cognitive impairment had lower DDS than the normal group (*P* < 0.001). After controlling for all covariates, DDS, physical activities, and cognitive functioning were negatively associated with all-cause mortality. Physical activities and cognitive function mediated 18.29% (95% confidence interval (CI) = 12.90–23.10) and 27.84% (95% CI = 17.52–37.56) of the total effect of DDS on mortality, respectively.

**Conclusions:**

Physical activities and cognitive function mediated the association between DDS and all-cause mortality. Maintaining DD may benefit early death prevention by reducing physical disability and cognitive impairment in community-dwelling older people.

In recent years, the number of older people globally has increased, and the ageing population in China has also shown a sustained upward trend [1]. Having the greatest proportion of older adults all over the world [2], the Chinese population aged ≥65 years had reached 158 million by the end of 2017, which was projected to expand to 366 million in 2050 [3]. With advancing age, older individuals experience a series of physiological changes, such as a decline in swallowing function, reduced taste sensitivity, slower gastrointestinal motility, decreased digestive and absorptive functions, and limitations in daily activities. Dietary diversity (DD) is defined as the number of different food groups consumed over a time period [4]. DD represents the variety of nutrient intake and is a component of the dietary quality and healthy dietary pattern [5,6]. Receiving an inadequately diversified diet may lead to undernutrition, which not only contributes to the occurrence of age-related diseases but can also, in severe cases, lead to mortality [7–9]. Diversified diets are positively related to better cognitive function, slower cognitive decline, and reduced risk of dementia [10], and they can reduce the risk of chronic diseases (cardiovascular and cerebrovascular diseases, etc.) [11]. Moreover, better DD helps maintain physical function and protects against the decline in multidimensional outcomes [12]. Therefore, enhancing the dietary quality of older people is crucial for achieving healthy ageing.

Various dietary guidelines [13–15] and studies [16,17] underscore the significance of a diverse, balanced diet and identified DD as a key indicator for evaluating dietary quality among older adults. However, a majority of studies have predominantly examined Western dietary patterns, which may not apply to older Chinese people [18,19]. The Dietary Diversity Score (DDS) is an effective metric for assessing the variety and inclusivity of foods consumed within a specified timeframe [20]. Currently, DDS is widely adopted for assessing DD levels in individuals. While DDS does not provide a comprehensive evaluation of an individual’s overall nutritional intake, it offers a practical means to assess adequacy [13], aligning with local dietary guidelines that emphasise diverse food group inclusion. Compared to the complex measurements of other dietary patterns, DDS is more straightforward and accessible for the oldest-old individuals, particularly in the cultural context of Chinese dietary habits [4,21,22]. Moreover, for older individuals, especially those with lower levels of education or cognitive impairments, the concise and quantitative DDS scale effectively evaluates dietary quality. Studies across diverse populations have consistently linked DDS with mortality risk among older adults [23–25]. Despite the existing literature supporting prolonged lifespan through DD, research on the underlying mechanisms remains sparse.

In previous research, physical activities and cognitive function have been considered as the two most crucial assessment indicators affecting the lifespan of older individuals [26,27]. Ageing is inherently associated with the loss of muscle mass, predisposing individuals to frailty, sarcopenia, and functional disability [28]. Notably, the ageing population requires increased dietary protein to maintain optimal muscle function [29]. Prospective studies have indicated that higher dairy product consumption correlates with a decreased risk of functional disability [30]. The diet quality of older individuals is closely related to physical function, with a higher DDS consistently associated with improved activities of daily living (ADL) [31], reduced sarcopenia risk, and enhanced bone health [32–36]. Furthermore, DDS exhibits a positive correlation with key indicators of physical performance, such as grip strength and gait speed [37], both of which are vital determinants of survival [38].

Various foods and nutrients exert distinct impacts on cognitive abilities. Certain dietary components and the synergistic effects of different food groups possess anti-inflammatory and antioxidant properties, influencing neuronal pathways and physiological mediators [39–42]. Studies suggested that the intake levels of specific food combinations and essential nutrients, including folate, vitamins, and minerals, are closely intertwined with the cognitive function of older adults [43,44]. Indeed, inadequate DD has been significantly associated with an elevated risk of cognitive impairment among older Chinese adults [45]. Additionally, meta-analysis evidence suggests that greater adherence to the Mediterranean diet is linked to a reduced risk of cognitive impairment and Alzheimer disease [46]. Furthermore, cohort studies have consistently reported that lower cognitive function measured in older populations is associated with increased total mortality rates [47–50]. Nevertheless, the precise roles of physical function and cognitive abilities in mediating the impact of DD on the mortality of older adults remain unclear.

Therefore, based on the close association between physical function, cognitive abilities, and DD, we propose that physical function and cognitive abilities may mediate the impact of DD on older adults’ mortality risk. In the current study, we validated this hypothesis using a large-scale cohort of older individuals residing in Chinese communities. Further, we explored the mechanisms of DD in healthy ageing.

## METHODS

### Study population

**Figure 1:**
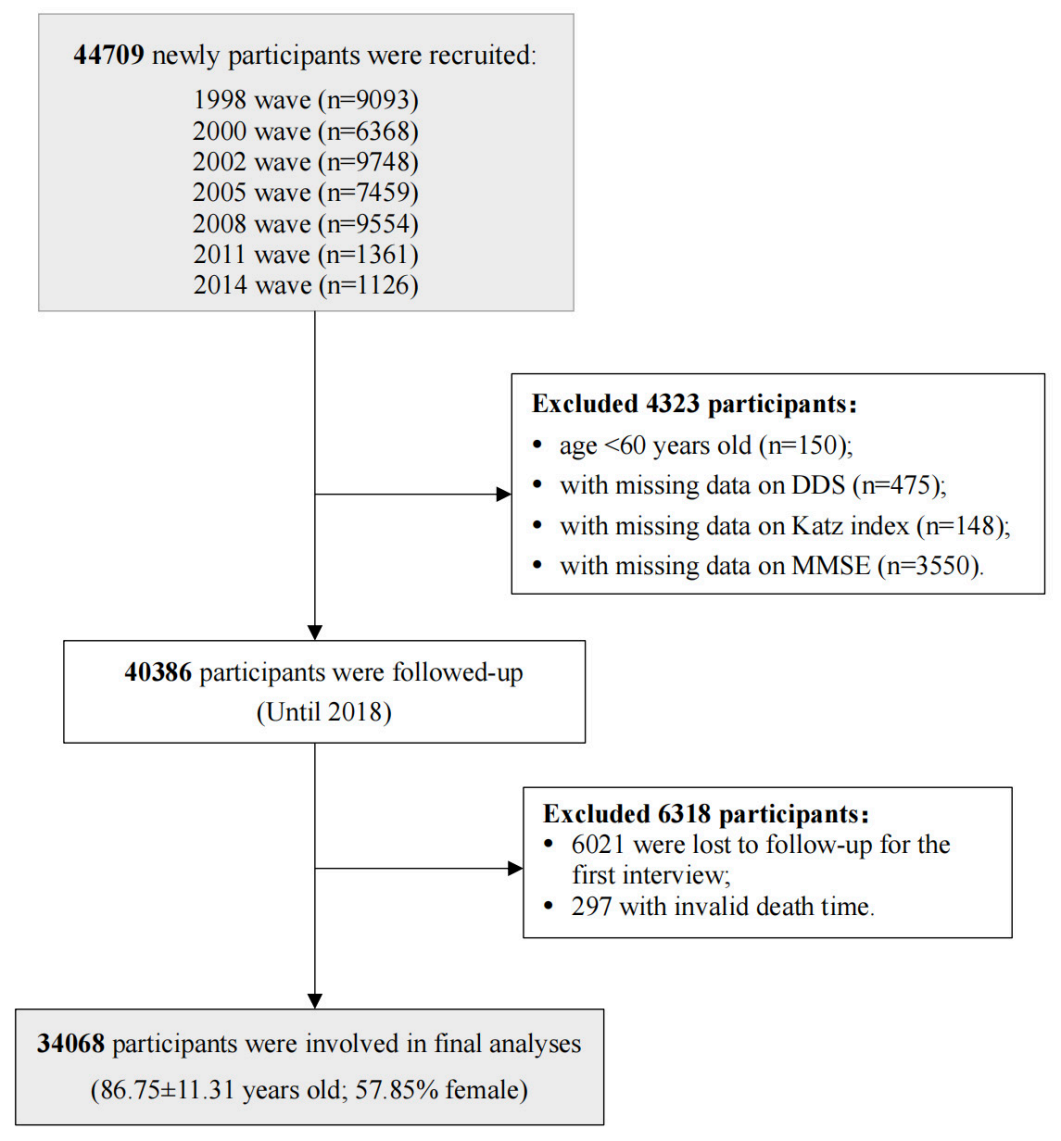
Flowchart of participant recruitment and follow-up interviews.

We used data from the Chinese Longitudinal Healthy Longevity Study (CLHLS), a national prospective cohort study that began in 1998 and was followed up every two to three years, with new participants enrolled at each follow-up [26]. Initially, 44 709 participants from seven waves (1998, 2000, 2002, 2005, 2008, 2011, and 2014) were recruited, and all participants were followed up until 2018. For instance, older individuals recruited in 1998 may have been followed up in the subsequent seven waves, whereas those newly added in 2014 would only be followed up in the 2018 wave. Baseline data were collected at the time when participants were enrolled. Questionnaires used in the current study remain almost consistent across each wave, ensuring the uniformity of the main variables and covariates. We sequentially excluded participants aged <60 years (n = 150), with missing data on DD (n = 475), physical activities (n = 148), and cognitive function (n = 3550). Besides, 6021 lost in the first follow-up, and 297 who had invalid death times were also excluded. 34 068 older adults were included in the final analysis (**Figure 1**). The CLHLS project was approved by the Biomedical Ethics Committee of Peking University (IRB00001052-13074), and all participants or their close relatives signed informed consent.

### Mortality and survival data

The time of death of deceased older people was obtained from the death registration system of the local civil affairs bureau or by asking close relatives. Participants who could not be contacted themselves or via their families were defined as censored, and the recorded survival time was the interval between the time of the last follow-up survey and enrolment. For participants who died, survival time was defined as the interval between the times of death and enrolment.

### Dietary diversity

We evaluated DDS using a simplified questionnaire including the frequency of intake of nine major food sources: fresh fruit, fresh vegetables, meat, fish, eggs, beans, tea, preserved vegetables, and garlic. The DD questionnaire is more straightforward to complete [6], and its validity and reproducibility for older Chinese adults have already been described in several previous studies [22,51–53]. We classified participants’ responses to the frequency of food consumption as frequently (*≥*5 times per week – three points), occasionally (1–4 times per week – two points) or rarely (<1 per week – one point). The overall DDS ranged from nine to 27. A higher DDS indicated a higher DD. After the Kolmogorov-Smirnov test, the total DDS was confirmed to be normally distributed (*P* = 0.175).

### Physical activities and cognitive function

We assessed physical activities using the Katz Index, which included six activity tasks of daily living (eating, dressing, toileting, bathing, transferring, and continence) [54]. Each task was answered with ‘need any assistance (complete independence or partial dependence) = 0’ and ‘complete dependence = 1’. In accordance with previous studies, participants with a total score <6 were classified as having a physical disability [55].

We assessed cognitive function using the Mini-Mental State Examination (MMSE) during face-to-face interviews. The MMSE includes 24 items, covering six dimensions (orientation, attention, language, registration, recall, and calculation). Answers ‘not able to answer’ and ‘wrong’ were recoded as zero points. MMSE score ranges from zero to 30, where higher global scores indicate better cognitive function. The validity of the Chinese version of MMSE has been confirmed [56]. Consistent with previous studies, illiterate subjects with an MMSE score <18 or literate subjects with an MMSE score <24 were defined as having cognitive impairment [57,58].

### Covariates

Due to the strong statistical power of this large-scale sample, we included a series of covariates to minimise the potential confounding. With reference to previous studies, the following covariates were corrected in the multiple Cox models: age, sex, ethnicity (Han or ethnic minorities), household registration (rural or urban), living alone (yes or no), level of education (illiterate or others), currently married (yes or no), occupation (farmer or others), financial support type (employed, retirement pension, and relative/community), smoking (yes or no), alcohol consumption (yes or no), regular exercise (yes or no), and body mass index (BMI). The number of natural teeth and use of dentures were collected during the face-to-face interviews by training investigators. History of disease, including hypertension, cerebrovascular disease, heart disease, respiratory disease, diabetes, and cancer, was collected through a standard self-report questionnaire.

### Statistical analysis

Data were presented as mean and standard deviation (SD) or n (%). We used Student’s *t* test, Wilcoxon rank sum test, or χ^2^ test to compare the baseline characteristics between participants with different follow-up outcomes. We used Spearman correlations to analyse the associations of DDS with physical activities and cognitive function. Further, we conducted restricted cubic spline regressions to examine the linear or nonlinear relationships among DDS, Katz index, MMSE, and all-cause mortality risk. Potential confounders were included in the adjusted Cox model, and hazard ratio (HR) and 95% confidence interval (CI) were calculated.

We conducted a causal mediation analysis based on a counterfactual framework to explore the mediating effects of physical activity and cognitive function on the relationship between DD and all-cause mortality [59,60]. In the mediation analysis framework, the total effect (presented as HR) of one unit (point) increase of DDS on mortality was decomposed into pure natural direct and indirect effects. Proportion mediated was used to evaluate the effect of each mediator.

We conducted the following sensitivity analyses to test the stability of the mediation results. First, considering the potential reverse causality due to early decedents, we sequentially excluded 3172 death events in the first year. Second, since severe pathological conditions may confound the relationship, we sequentially excluded participants who had heart disease (n = 2568), cerebrovascular disease (n = 1456), or cancer (n = 419) at baseline. All statistical analyses were performed using R, version 4.2.0 (R Core Team, Vienna, Austria) in which the mediation analysis was conducted using package ‘regmedint’, and a two-tailed *P*-value <0.05 was considered significant.

## RESULTS

### Sample characteristics

The age of all participants at baseline ranged from 60–114 years, with a mean age of 86.75 (SD = 11.31), and 57.85% were female. The mean score of DDS was 18.72 (SD = 2.97) (**Table 1**). During 148 195.58 person-years of follow-up, 25 362 older adults died. Participants who had low DDS, Katz index score, and MMSE score were more likely to die (*P* < 0.001). We found DDS was positively correlated with Katz index score (*r* = 0.128, *P* < 0.001) and MMSE score (*r* = 0.189, *P* < 0.001) (Table S1 in the **Online Supplementary Document**). Among all participants, there were 9030 (26.51%) older individuals with physical disability and 7283 (21.38%) with cognitive impairment. Participants who had a physical disability or cognitive impairment showed lower DDS (*P* < 0.001).

**Table 1:** Demographic characteristics of 34 086 older adults across follow-up outcomes*

Characteristics	Overall (n = 34 068)	Survived (n = 8706)	Deceased (n = 25 362)	*P*-value†
Age in years, x̄ (SD)	86.75 (11.31)	78.94 (10.93)	89.78 (9.73)	<0.001
Female	19 709 (57.85)	4707 (54.07)	15 002 (59.15)	<0.001
Han ethnic	31 707 (93.35)	8190 (94.62)	23 517 (92.91)	<0.001
Rural	26 112 (76.65)	6237 (71.64)	19 875 (78.37)	<0.001
Live alone	4400 (12.92)	1249 (14.36)	3135 (12.43)	<0.001
Less than one year of schooling	21 971 (65.76)	4463 (53.16)	17 508 (69.99)	<0.001
Currently married	9342(27.42)	4262(48.95)	5080(20.03)	<0.001
Farmer	21 438 (62.93)	5092 (58.49)	16 346 (64.45)	<0.001
Financial support				<0.001
*Working/employed*	2551 (7.49)	1445 (16.60)	1106 (4.36)	
*Retirement wage*	5374 (15.77)	2210 (25.38)	3164 (12.48)	
*Relative or community*	26 143 (76.74)	5051 (58.02)	21 092 (83.16)	
BMI in kg/m^2^, x̄ (SD)	19.59 (4.08)	20.97 (4.03)	19.11 (3.99)	<0.001
Smoking	6365 (18.69)	1858 (21.35)	4507 (17.77)	<0.001
Alcohol consumption	7178 (21.08)	1912 (21.98)	5266 (20.77)	0.018
Regular exercise	9545 (28.05)	3215 (37.02)	6330 (24.98)	<0.001
Number of teeth				<0.001
*0*	12303 (36.11)	2186 (25.11)	10 117 (39.89)	
*1*–*10*	12 565 (36.88)	2508 (28.88)	10 057 (39.65)	
*11*–*36*	9200 (27.01)	4012 (46.08)	5188 (20.46)	
Denture use	8183 (24.02)	2768 (31.79)	5415 (23.18)	<0.001
DDS, x̄ (SD)	18.72 (2.97)	19.29 (2.91)	18.32 (2.97)	<0.001
Katz index score, x̄ (SD)	5.29 (1.45)	5.81 (0.69)	5.11 (1.65)	<0.001
MMSE, x̄ (SD)	20.75 (6.30)	25.25 (6.01)	19.20 (6.72)	<0.001
Hypertension	5244 (15.51)	1737 (20.12)	3507 (13.93)	<0.001
Diabetes	570 (1.69)	222 (2.57)	348 (1.38)	<0.001
Heart disease	2568 (7.59)	824 (9.54)	1744 (6.92)	<0.001
Cerebrovascular disease	1456 (4.30)	386 (4.47)	1070 (4.25)	0.388
Respiratory disease	3789 (11.20)	860 (9.97)	2927 (11.62)	<0.001
Cancer	419 (1.23)	124 (1.42)	295 (1.16)	0.061

BMI – body mass index, DDS – dietary diversity score, MMSE – Mini-Mental State Examination, x̄ – mean, SD – standard deviation

*Presented as n (%) unless specified otherwise.

†*P*-values were generated using the Student’s *t*-test, Wilcoxon rank sum test, or χ^2^ test.

### Associations of DDS, physical activities, and cognitive function with mortality

**Figure 2:**
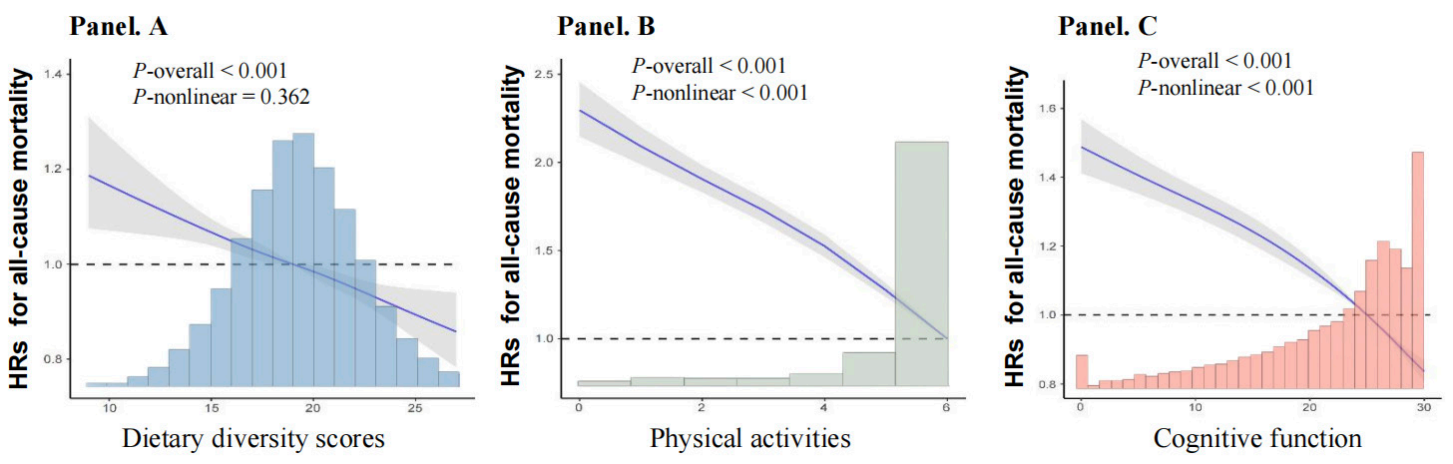
Dose-response relationship between exposures and all-cause mortality. Adjusted for age and sex, ethnicity, residence, living status, education, marital status, occupation, financial support, body mass index, smoking, drinking, regular exercise, number of teeth, denture use, hypertension, diabetes, heart disease, cerebrovascular disease, respiratory disease, and cancer. **Panel A.** Association between dietary diversity scores and all-cause mortality. **Panel B.** Association between physical activities. and all-cause mortality. **Panel C.** Association between cognitive function and all-cause mortality.

After adjusting for age, sex, ethnicity, residence, living status, education, marital status, occupation, financial support, BMI, smoking, drinking, regular exercise, number of teeth, denture use, and disease history, DDS was linearly negatively associated with all-cause mortality (*P* = 0.362). We observed nonlinearly negative associations of physical activity and cognitive function with all-cause mortality risk (*P* < 0.001) in multiple restricted cubic spline regressions (**Figure 2**).

### Mediation analysis

**Figure 3:**
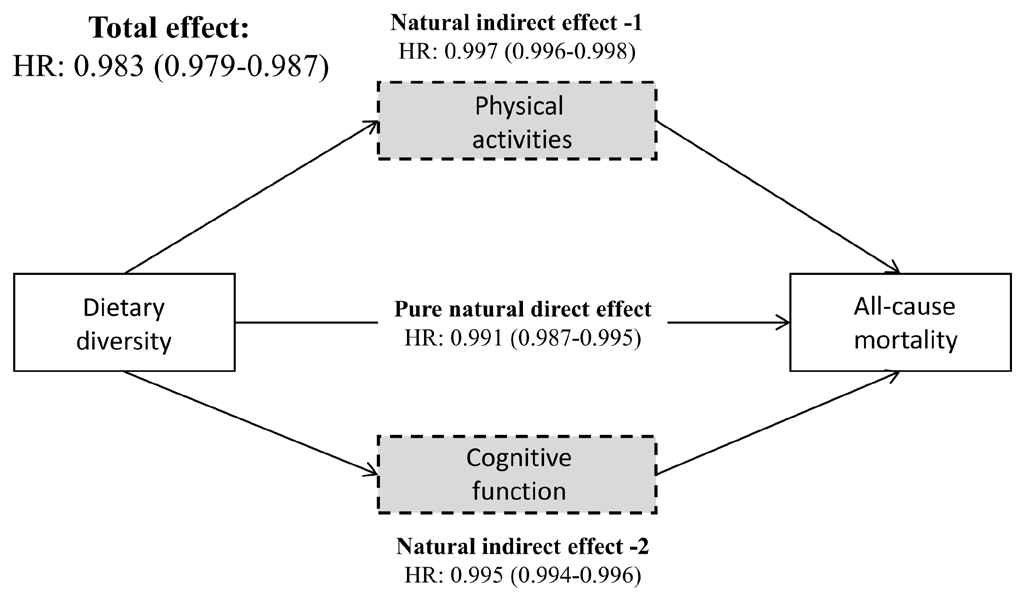
Mediating roles of physical activities and cognitive function in the relationship between dietary diversity and all-cause mortality. Adjusted for age and sex, ethnicity, residence, living status, education, marital status, occupation, financial support, body mass index, smoking, drinking, regular exercise, number of teeth, denture use, hypertension, diabetes, heart disease, cerebrovascular disease, respiratory disease, and cancer.

DDS, physical activity, and cognitive function were included as continuous variables in the mediation analysis, and all HRs in the mediation model were statistically significant after adjusting for all covariates. The total effect and pure natural direct effect of DDS on mortality were HR = 0.983 (95% CI = 0.979–0.987) and HR = 0.991 (95% CI = 0.987–0.995), respectively. The indirect effect mediated by physical activity and cognitive function was HR = 0.997 (95% CI = 0.996–0.998) and HR = 0.995 (95% CI = 0.994–0.996). Thus, the proportion mediated for physical activity and cognitive function in the relationship between DDS and all-cause mortality were 18.29% (95% CI = 12.90–23.10) and 27.84% (95% CI = 17.52–37.56) (**Figure 3**). In sex-stratified analyses, we found the mediating effect of physical activity and cognitive function was more pronounced in women than in men (**Table 2**).

**Table 2:** Causal mediating effects of physical activities and cognitive function on the association between dietary diversity and all-cause mortality stratified by sex*

Items	Overall (n = 34 068)	*P*-value	Men (n = 14 359)	*P*-value	Women (n = 19 709)	*P*-value
Death, n (%)	25 362 (74.45)		10 360 (72.15)		15 002 (76.12)	
Person-years	148 188.03		66 255.90		81 932.13	
Total effect, HR (95% CI)	0.983 (0.979–0.987)	<0.001	0.977 (0.972–0.982)	<0.001	0.985 (0.978–0.991)	<0.001
Pure natural direct effect, HR (95% CI)	0.991 (0.987–0.995)	<0.001	0.987 (0.983–0.991)	<0.001	0.993 (0.988–0.997)	<0.001
Indirect effect by PA, HR (95% CI)	0.997 (0.996–0.998)	<0.001	0.996 (0.995–0.997)	<0.001	0.996 (0.995–0.997)	<0.001
*Proportion mediated, % (95% CI)*	18.29 (12.90–23.10)		15.85 (9.97–21.05)		23.46 (13.04–33.32)	
Indirect effect by CF, HR (95% CI)	0.995 (0.994–0.996)	<0.001	0.994 (0.993–0.995)	<0.001	0.995 (0.994–0.996)	<0.001
*Proportion mediated, % (95% CI)*	27.84 (17.52–37.56)		26.50 (15.88–36.32)		30.47 (18.18–42.77)	

CF – cognitive function, CI – confidence interval, HR – hazard ratio, PA – physical activity

*Adjusted for age and sex, ethnicity, residence, living status, education, marital status, occupation, financial support, BMI, smoking, drinking, regular exercise, number of teeth, denture use, hypertension, diabetes, heart disease, cerebrovascular disease, respiratory disease, and cancer.

### Sensitivity analyses

All effects in the sensitivity analyses remained significant. We found that excluding participants who died in the first follow-up year did not appreciably attenuate the mediating effect of physical activity and cognitive function on the relationship between DDS and mortality (Table S2 in the **Online Supplementary Document**). Additionally, when we excluded participants with heart disease, cerebrovascular disease, or cancer at baseline, the main results were materially unchanged (Tables S3–5 in the **Online Supplementary Document**).

## DISCUSSION

In this large population-based longitudinal study of older adults, we examined the mediating roles of physical activities and cognitive function in the relationship between DD and all-cause mortality. To our knowledge, this is the first cohort study in a nationally representative sample of Chinese older adults, indicating that both physical activity and cognitive function play crucial mediating roles in the association between DDS and mortality risk in this population. Our findings reveal that physical activity and cognitive function contribute 18.29% and 27.84% of the total effect, respectively, with a more pronounced mediating effect in women than in men.

This study established an inverse association between DDS and mortality risk among the ageing population, aligning with previous studies. Cohort studies among Chinese older adults consistently validated this inverse relationship [51,61], echoed by longitudinal findings in Thailand emphasising a pronounced effect among older individuals [62]. In Japan, a comprehensive cohort study over an extended follow-up period demonstrates DDS’s inverse associations with overall mortality and cancer-related mortality. However, no significant links were found with mortality related to cardiovascular and cerebrovascular events [63]. Conversely, a study involving French older adults did not find significant associations between DDS and mortality [64]. The Japan Public Health Centre-based prospective study indicates an inverse association between DDS and all-cause as well as cardiovascular disease mortality, particularly in women within the highest quintile, while no such associations were observed in men [65]. Inconsistencies across studies may stem from sample size limitation, unidentified confounding factors, or selection bias due to low response rates. Additionally, the lack of standardised tool to measure DD and variations in food group inclusion might contribute to differing relationships between DD and health outcomes.

Our study employs a robust index of DDS primarily composed of healthy food items, emphasising plant-based and antioxidant-rich foods like fruits, vegetables, and fish. These dietary components are crucial as they are associated with cognitive health benefits and anti-inflammatory effects [66–70]. High-quality protein sources such as meat, eggs, bean products and milk products are essential for muscle protein metabolism in the older population [35], and are also included in our index. Thus, our DDS may indicate higher diet quality scores, assessing adequate food consumption and diet healthfulness [71].

Ageing is multifactorial, involving challenges such as insufficient protein intake, chronic inflammation, and inadequate vitamin and mineral levels, highlighting the significance of increased DDS for improved survival. There are several ways in which an increased DDS might improve survival. First, DDS serves as a proxy for nutrient intake [72], crucial for maintaining better health in older adults based on numerous guidelines [73]. Adequate and varied nutrient intake can enhance health status and longevity [74]. Second, the balance and interaction of multiple nutrients within the body are facilitated by rich DD, supporting overall health [75]. Third, higher DDS correlates with a diverse and healthier gut microbiota pattern [76], linked to reduced inflammation and lower risks of chronic diseases prevalent in older adults [77,78]. Moreover, this study underscores the roles of inflammation and oxidative stress in ageing, highlighting the anti-inflammatory and antioxidant effects of DD on brain health [79]. Reduced DD may exacerbate oxidative damage [80], with studies indicating associations between oxidative stress and increased risk of all-cause mortality among older adults [81]. Additionally, higher DDS scores are associated with greater plasma magnesium levels, further linked to lower mortality risks [82]. These findings underscore the potential biological mechanisms underlying the observed relationships and emphasise the implications for public health interventions promoting healthy ageing beyond specific dietary components.

Our findings reveal a positive correlation between DDS and physical activity (*r* = 0.128, *P* < 0.001), as well as cognitive function (*r* = 0.189, *P* < 0.001), aligning with previous research. Higher DDS had heightened intakes of protein, which may mitigate age-related muscle loss and neuromuscular decline, thereby potentially preventing or delaying sarcopenia and frailty [83,84]. This dietary pattern also supports optimal muscle function in older adults [83,85,86]. In addition, participants with higher DDS tend to consume more calcium, phosphorous, and potassium, which contribute to better bone health [87]. Adequate calcium and vitamin D intake may reduce the risk of osteoporosis-related impairments in physical function and fractures, along with maintaining muscle strength [88]. Moreover, antioxidants found abundantly in fruits, vegetables, mushrooms, algae, tea, and garlic – such as vitamin C and carotene – are associated with enhanced skeletal muscle strength, potentially mitigating inflammation and oxidative stress, thus preserving physical function [89]. Finally, higher DD reduces the likelihood of exposure to harmful substances in food and supports telomere length maintenance, which is linked to sustained muscle strength [90–92].

Importantly, cognitive function contributed more significantly to the mediated effect than physical activity (27.84% vs 18.29%), suggesting that DDS’s beneficial impact on mortality primarily operates through its role in mitigating central nervous system degeneration [93]. Previous studies have indicated that higher protein consumption may protect cognitive function among the Chinese oldest [94], as well as in older populations in Canada [95], France [96] and Australia (mean age = 60 years) [97]. DD also correlates positively with microbiome stability, potentially influencing brain function via the brain-gut-microbiome Axis [98,99]. Furthermore, the varied components of a diverse diet could enhance cognitive function by influencing synaptic plasticity and membrane fluidity [100]. Given that ageing brains are susceptible to oxidative damage and often exhibit poor antioxidant status [41,101], our findings suggest that DD may offer protective benefits, particularly to cognitive health [102]. Otsuka et al. similarly found that DD positively influenced higher-level functional capacities among older adults [103].

Gender-specific analyses reveal a more pronounced mediating effect of physical activity and cognitive function in women, possibly due to their higher prevalence in this population [79,104]. Moreover, the bio-physiological sensitivity of dietary intake may differ between genders [105]. Consistent with our findings, cross-sectional studies have linked higher DDS with reduced risks of cognitive impairment among older Chinese women [106,107]. Similarly, studies have shown that mild cognitive impairment was inversely related to the dietary pattern scores [108], particularly in women, and that malnutrition adversely affects cognitive function, especially among the oldest, less educated females in China [109]. In contrast, Lv et al. observed a more significant protective effect of DDS on mortality in men, possibly explained by men’s greater capability for nutrient digestion and absorption [51]. Notably, similarities in dietary patterns across sexes suggest that differences in the diet-cognition relationship may arise from varying impacts on cognitive function in later life rather than differences in food consumption per se [110,111]. Furthermore, higher educational levels are associated with greater cognitive reserves, potentially compensating for brain damage and preserving cognitive function [112]. These gender-specific differences underscore the need for further exploration into how DD influences physical activity, cognitive function, and ultimately mortality risk, and suggest that consuming a greater diversity of total foods may have considerable positive public health implications for both men and women.

Strengths of our study include its nationally representative, population-based sample with a long period of follow-up and a large sample size, covering 23 provinces or municipalities in China. We filled previous knowledge gaps by examining the association between DDS and mortality among older Chinese adults within a counterfactual framework, elucidating the mediating roles of physical function and cognitive abilities [113]. However, several methodological limitations are considered. First, dietary intake data relied on self-reported frequencies via food frequency questionnaires, which may be susceptible to recall bias. In addition, although the validity of the CLHLS dietary questionnaire has been confirmed, it is unable to quantify food intake. Second, although we adjusted for many potential confounding factors, we cannot rule out the effects of residual and unmeasured confounding in this observational study. However, sensitivity analyses excluding specific chronic conditions showed stable results, and future research needs to consider the potential confounding effects of undiagnosed diseases. Third, DDS was measured at a single time point when participants were enrolled, which is limited for precise causal inference. The effect of DD on health accumulates across the lifespan. Thus, future longitudinal research should focus on the effects of the dynamic changes in DDS on health outcomes and mortality. Fourth, our findings may be influenced by survival bias or selection bias inherent in studies of older populations. Individuals nearing 80 years may modify their lifestyles due to declining health [114], potentially diluting the observed effects of DDS on mortality. Moreover, the focus on long-lived individuals in CLHLS [115] could introduce healthier survivor bias, where those with healthier dietary behaviours are overrepresented due to longer survival [116], thereby underestimating the true effects of DDS among older adults. Nevertheless, sensitivity analyses, including the exclusion of early deaths and prevalent cases of severe conditions, mitigate concerns regarding reverse causation and selection biases to some extent. Future research efforts should aim to overcome these limitations through more precise dietary assessment methods and rigorous study designs to further elucidate the impacts of DD on health outcomes in the ageing population.

## CONCLUSIONS

In the older Chinese population, we identified physical activity and cognitive function as significant mediators in the association between DDS and all-cause mortality. This underscores DDS’s potential as a straightforward tool for assessing mortality risk among older individuals, highlighting its role in mitigating early death through the preservation of physical and cognitive health. Ultimately, our findings provide compelling evidence supporting the promotion of healthy ageing through a well-balanced and diverse diet. These insights advocate for public health strategies aimed at encouraging DD to enhance longevity and quality of life in the ageing population.

## Additional material


Online Supplementary Document
