# Supplementary material for: Mediating effects of physical activities and cognitive function on the relationship between dietary diversity and all-cause mortality in community-dwelling older adults
Source: J Glob Health. 2024 Oct 25;14:04169. doi: 10.7189/jogh.14.04169 (PMC11505579; doi:10.7189/jogh.14.04169)
Supplement: Online Supplementary Document [file jogh-14-04169-s001.pdf]

Supplementary materials

Supplementary table 1. Correlations among dietary diversity score, Katz index score, and Mini-Mental State Examination

**Variables**	Dietary diversity score	Katz index score	Mini-Mental State Examination score
Dietary diversity score	1		
Katz index score	0.128***	1	
Mini-Mental State Examination score	0.189***	0.504***	1

*** Spearman correlation coefficients P<0.001

Supplementary table 2 (Sensitivity analysis 1). The mediating role of physical activities and cognitive function in the relationship between dietary diversity and all-cause mortality.

Excluding 3172 decedents in the first year, 30896 were included.

Pathways	HR (95%CI)	*P*-value	Proportion mediated, % (95%CI)
**Total effect**	0.986 (0.983-0.989)	<0.001	
**Pure natural direct effect**	0.992 (0.988-0.996)	<0.001	
**Indirect effect-** by physical activities	0.998 (0.996-0.999)	<0.001	14.29 (10.33-18.25)
**Indirect effect-** by cognitive function	0.996 (0.994-0.998)	<0.001	28.57 (19.61-37.53)

Notes: Adjusted for age and sex, ethnicity, residence, living status, education, marital status, occupation, financial support, BMI, smoking, drinking, regular exercise, number of teeth, denture use, hypertension, diabetes, heart disease, cerebrovascular disease, respiratory disease, and cancer.

Supplementary table 3 (Sensitivity analysis 2). The mediating role of physical activities and cognitive function in the relationship between dietary diversity and all-cause mortality.

Excluding 2568 participants with heart disease, 31500 were included.

Pathways	HR (95%CI)	*P*-value	Proportion mediated, % (95%CI)
**Total effect**	0.983 (0.980-0.986)	<0.001	
**Pure natural direct effect**	0.991 (0.987-0.995)	<0.001	
**Indirect effect-** by physical activities	0.997 (0.995-0.999)	<0.001	17.65 (13.69-21.61)
**Indirect effect-** by cognitive function	0.995 (0.993-0.997)	<0.001	29.41 (20.45-38.37)

Notes: Adjusted for age and sex, ethnicity, residence, living status, education, marital status, occupation, financial support, BMI, smoking, drinking, regular exercise, number of teeth, denture use, hypertension, diabetes, cerebrovascular disease, respiratory disease, and cancer.

Supplementary table 4 (Sensitivity analysis 3). The mediating role of physical activities and cognitive function in the relationship between dietary diversity and all-cause mortality.

Excluding 1456 participants with heart disease, 32612 were included.

Pathways	HR (95%CI)	*P*-value	Proportion mediated, % (95%CI)
**Total effect**	0.981 (0.978-0.984)	<0.001	
**Pure natural direct effect**	0.989 (0.985-0.993)	<0.001	
**Indirect effect-** by physical activities	0.997 (0.995-0.999)	<0.001	15.79 (11.83-19.75)
**Indirect effect-** by cognitive function	0.995 (0.993-0.997)	<0.001	26.32 (17.36-35.28)

Notes: Adjusted for age and sex, ethnicity, residence, living status, education, marital status, occupation, financial support, BMI, smoking, drinking, regular exercise, number of teeth, denture use, hypertension, diabetes, heart disease, respiratory disease, and cancer.

Supplementary table 5 (Sensitivity analysis 4). The mediating role of physical activities and cognitive function in the relationship between dietary diversity and all-cause mortality.

Excluding 419 participants with cancer, 33649 were included.

Pathways	HR (95%CI)	*P*-value	Proportion mediated, % (95%CI)
**Total effect**	0.983 (0.980-0.986)	<0.001	
**Pure natural direct effect**	0.992 (0.988-0.996)	<0.001	
**Indirect effect-** by physical activities	0.996 (0.994-0.998)	<0.001	22.81 (18.85-26.77)
**Indirect effect-** by cognitive function	0.995 (0.993-0.997)	<0.001	30.41 (21.45-39.37)

Notes: Adjusted for age and sex, ethnicity, residence, living status, education, marital status, occupation, financial support, BMI, smoking, drinking, regular exercise, number of teeth, denture use, hypertension, diabetes, heart disease, cerebrovascular disease, and respiratory disease.
